# Residual Spatial and Channel Attention Networks for Single Image Dehazing

**DOI:** 10.3390/s21237922

**Published:** 2021-11-27

**Authors:** Xin Jiang, Chunlei Zhao, Ming Zhu, Zhicheng Hao, Wen Gao

**Affiliations:** 1 Changchun Institute of Optics, Fine Mechanics and Physics, Chinese Academy of Sciences, Changchun 130033, China; zhaochunlei@ciomp.ac.cn (C.Z.); zhuming@ciomp.ac.cn (M.Z.); haozhicheng@ciomp.ac.cn (Z.H.); gaowen@ciomp.ac.cn (W.G.); 2 University of Chinese Academy of Sciences, Beijing 100049, China

**Keywords:** single image dehazing, residual spatial and channel attention network, conditional generative adversarial network, dense network, contrastive loss, registration loss

## Abstract

Single image dehazing is a highly challenging ill-posed problem. Existing methods including both prior-based and learning-based heavily rely on the conceptual simplified atmospheric scattering model by estimating the so-called medium transmission map and atmospheric light. However, the formation of haze in the real world is much more complicated and inaccurate estimations further degrade the dehazing performance with color distortion, artifacts and insufficient haze removal. Moreover, most dehazing networks treat spatial-wise and channel-wise features equally, but haze is practically unevenly distributed across an image, thus regions with different haze concentrations require different attentions. To solve these problems, we propose an end-to-end trainable densely connected residual spatial and channel attention network based on the conditional generative adversarial framework to directly restore a haze-free image from an input hazy image, without explicitly estimation of any atmospheric scattering parameters. Specifically, a novel residual attention module is proposed by combining spatial attention and channel attention mechanism, which could adaptively recalibrate spatial-wise and channel-wise feature weights by considering interdependencies among spatial and channel information. Such a mechanism allows the network to concentrate on more useful pixels and channels. Meanwhile, the dense network can maximize the information flow along features from different levels to encourage feature reuse and strengthen feature propagation. In addition, the network is trained with a multi-loss function, in which contrastive loss and registration loss are novel refined to restore sharper structures and ensure better visual quality. Experimental results demonstrate that the proposed method achieves the state-of-the-art performance on both public synthetic datasets and real-world images with more visually pleasing dehazed results.

## 1. Introduction

In recent years, hazy weather has become increasingly frequent, which seriously affects our daily production and life. Haze is a natural phenomenon caused by the absorption of scattered light by particles in the atmosphere [1]. Under such conditions, optical equipments are not able to obtain effective scene information with poor image quality, which severely limits the subsequent image processing in satellite remote sensing, video monitoring, automatic driving and other fields; therefore, the question of how to effectively remove haze across an image, restore color and contrast of the image as much as possible without losing details or introducing additional interference information is of important research significance.

Single image dehazing is a challenging problem, which has attracted extensive attention from academia and industry [2,3,4,5]. Single image dehazing refers to the methods of restoring clear and natural images with recognizable details and abundant color from input hazy images that are taken under hazy weather conditions [6]. Some existing dehazing methods including both prior-based and learning-based heavily rely on the simplified atmospheric scattering model, which can be formulated as

(1) $$I\left(x\right)=J\left(x\right)t\left(x\right)+A\left(1-t(x)\right),$$

where I(x) and J(x) denote the hazy and corresponding haze-free images, respectively. t(x) denotes the medium transmission map, and *A* is the global atmospheric light. t(x) can be further expressed as

(2) $$t\left(x\right)=e^{-\beta d(x)},$$

where d(x) denotes the depth of scene point and β is defined as the scattering coefficient of atmosphere. Based on this, the methods firstly estimate the transmission map and global ambient light as medium with the help of haze relevant characteristics or deep neural network, and then reconstruct haze-free images with a linear formula. These methods are often effective in some certain scenarios, but fail to remove haze in other complex scenarios, since they face the following technical difficulties: (i) The degradation process in reality is much more complicated and it is unreasonable to be described by a simple mathematical formula. (ii) The feature extracted from hazy images is too simple to cover complex scenarios. (iii) Different atmospheric scattering parameters affect each other in optimization and it is difficult to achieve global optimal, which further reduces the performance of haze removal. In addition, some learning-based dehazing methods treat spatial-wise and channel-wise features equally, lacking discriminative learning ability across different feature channels and pixels, since haze is practically unevenly distributed across an image, which greatly limits the representational ability of deep neural network.

To overcome these weaknesses, inspired by the significant performance of conditional generative adversarial network [7] on image-to-image translation problems, we propose a densely connected residual spatial and channel attention network bypassing the step of estimating atmospheric scattering parameters, which can directly generate a clear image from an input hazy image. Moreover, a novel residual attention module, which combines spatial attention and channel attention mechanism, is proposed. The module could adaptively rescale features by considering interdependencies among spatial and channel information, which would expand the representational ability of deep convolutional neural network, and allow the network to concentrate on more useful pixels and channels.

Our main contributions can be summarized as follows:

- We propose an end-to-end trainable network based on conditional generative adversarial architecture to solve the ill-posed single image dehazing problem. The network does not rely on the classical atmospheric scattering model, while adopts the method of image-to-image translation alternatively.
- An efficient module, called residual spatial and channel attention module, is designed to improve the ability of feature representation by adaptively recalibrating spatial-wise and channel-wise feature weights based on interdependencies among spatial and channel information, since haze is unevenly distributed across an image.
- A densely connected network in which feature maps are used as inputs to all subsequent layers, is derived to enhance reusability of features and transmission ability.
- Our method enhances conditional generative adversarial formulation by introducing novel refined contrastive loss and registration loss functions in order to better preserve the details, reduce artifacts and generate more visually pleasing images.
- Experiments evaluated on both public synthetic datasets and real-world images reveal that the proposed method achieves state-of-the-art single image dehazing methods in terms of both quantitative and visual performance.

The rest of this paper is organized as follows: In Section 2, we provide a brief overview of the related work. In Section 3, the detailed proposed architecture is presented. Experimental results are given and discussed in Section 4. Finally, the conclusion of this paper is given in Section 5.

## 2. Related Work

Single image dehazing and generative adversarial networks are the two topics related to this paper. In what follows, we provide a brief overview of these related works.

### 2.1. Single Image Dehazing

In recent years, a large number of single image dehazing methods has been proposed to solve this ill-posed problem. These methods can be roughly divided into two categories: prior-based methods and learning-based methods. Prior-based dehazing methods utilize manually designed priors or conjecture based on the atmospheric scattering model to carry out haze-free images. Learning-based dehazing methods make use of large datasets of hazy and haze-free images and powerful feature representation ability to realize efficient image dehazing.

By comparing hazy images with haze-free images, Tan et al. [8] observed that hazy images have lower contrast and sharpness. Meanwhile, the change of atmospheric light value in hazy images mainly depends on the distance between the object and the observer, the larger the distance, the smoother the change rate of atmospheric light value. Based on these, a Markov model is established to improve the local contrast of hazy images to achieve haze removal, but this method is prone to the problem of large color difference. Ancuti et al. [9] put forward a new concept, namely semi-inverse, which allows for fast identification of hazy regions. Based on the hue disparity between the input hazy image and its semi-inverse, they are able to identify hazy regions on a pixel-wise manner. After analyzing a large number of clear outdoor images, He et al. [10] proposed the classical dark channel prior: In the vast majority of non-sky areas, there always exists some pixels that have very low intensities in at least one color channel. Based on this, clear images are deduced by estimating medium transmission map and atmospheric light from the atmospheric scattering model. This method achieves better dehazing performance under certain conditions, but it fails in high brightness area such as sky regions. Similar to dark channel algorithm, color attenuation prior is also a statistical method in essence. Zhu et al. [11] found that haze concentration is positively proportional to the difference between brightness and saturation on the basis of analyzing a large number of images. With this prior, haze can be effectively removed by estimating the transmission and restoring the scene radiance via atmospheric scattering model. Berman et al. [12] remarked that pixels in a given cluster spread over all the image plane and are located at different distances from the observer. Both distance maps and haze-free images can be recovered with the help of so-called haze-lines. Wang et al. [13] derived a fast single image dehazing algorithm based on the linear transformation by considering that a linear relationship exists in the minimum channel between hazy image and its corresponding haze-free counterpart.

With the rapid development of deep learning, a large number of end-to-end deep neural networks has emerged in the field of image dehazing. Cai et al. [14] first introduced convolutional neural network into image dehazing task and proposed an end-to-end trainable dehazing network, which utilizes multi-scale convolution operations to extract haze features by taking hazy images as input and transmission maps as output. The atmospheric scattering model is applied to recover haze-free images, which greatly improves the haze removal performance compared with traditional methods. Li et al. [15] proposed a lightweight image dehazing network called AOD-Net, which does not estimate intermediate variables separately, but integrates multiple intermediate variables into one parameter through the identity transformation of the formula to minimize reconstruction error, and effectively improves the quality of recovered images. Ren et al. [16] designed an end-to-end threshold fusion dehazing network by adding some image preprocessing methods, which mainly includes white balance, contrast enhancement and gamma correction. The corresponding haze-free images are obtained based on the pixel-wise confidence maps. Ha et al. [17] proposed a novel residual-based single image dehazing method by adopting the gate fusion network in order to overcome the limitation caused by atmospheric scattering model-based methods. Qin et al. [18] proposed a feature fusion attention network to directly reconstruct haze-free images, in which a novel feature attention module is designed to pay more attention to the effective information such as thick haze regions. Kuanar et al. [19] developed a learning-based deglow–dehaze iterative network accounting for varying colors and glows, in order to address the single image haze removal problem in nighttime scenes. Shin et al. [20] present a dehazing and verifying network called DVNet and a correction network called CNet by directly estimating the radiance of hazy images with a self-supervised learning method.

### 2.2. Generative Adversarial Networks

Generative adversarial network (GAN) [21] is a neural network model based on the zero-sum game theory, which skillfully utilizes the adversarial idea to learn data distribution and generate new samples. GAN is mainly composed of a generator and a discriminator, in which the generator takes noises as input and generate new samples, while the discriminator receives and authenticates the authenticity of the generated samples and real samples. In the process of training, the generator aims to generate samples similar with target domain to fool the discriminator, while the discriminator’s goal is to try to distinguish generated samples from real samples.

Generative adversarial network is able to theoretically achieve the fitting of real data by distributing direct sampling, but it is prone to the problem of model collapse in face of images, leading to training failure and unsatisfactory generated samples. To remedy this problem, Mirza [7] proposed a conditional generative adversarial network (cGAN) by adding some constraints to the original GAN architecture. These additional constraint information performs a certain guiding effect on the generation of data, enhances the stability of training process, improves the representation ability of the generator and also successfully transforms unsupervised training into supervised training. This simple and direct improvement is very effective, and cGAN has been widely used in image haze removal [22,23,24], image rain removal [25,26,27] and other image generation fields [28,29,30].

## 3. Proposed Method

In this section, we present detailed architecture of the proposed densely connected residual spatial and channel attention network. First, we give an overview of the proposed network. Second, we introduce details of the generator module, the residual spatial and channel attention module and the discriminator module. Then we provide multi-loss functions for training the network.

### 3.1. Overview of the Proposed Architecture

**Figure 1:**
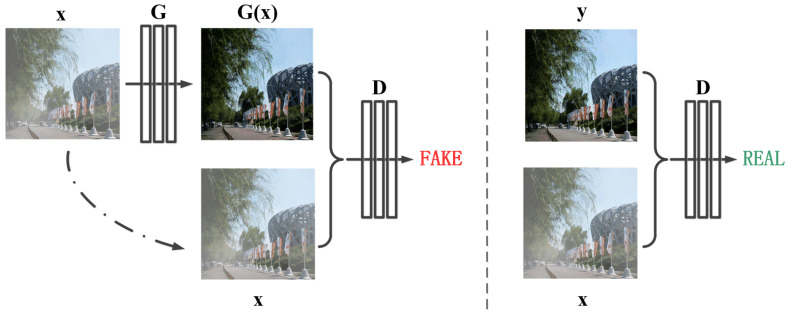
The architecture of the proposed framework. “G” denotes the generator and “D” denotes the discriminator. “x” is the input hazy image, “G(x)” is the reconstructed hazy-free image and “y” is the clear image. Unlike the unconditional GAN framework, both the generator and discriminator observe the input hazy image.

Inspired by the recent success of conditional generative adversarial network for pixel-to-pixel vision tasks, we aim to directly learn a mapping function from an input hazy image to a haze-free image by reconstructing a conditional GAN-based network. As shown in Figure 1, the proposed architecture is composed of a generator G and a discriminator D. The generator is constructed using the densely connected network [31] with residual spatial and channel attention module, which aims to restore hazy images from hazy domain to clear domain. The discriminator adopts efficient PatchGAN framework as used in pix2pix [32], and it is designed to classify whether the reconstructed images are clear or hazy. In what follows, we introduce the generator, residual spatial and channel attention module and discriminator in detail.

### 3.2. Generator

The goal of the generator is to directly reconstruct a clear image from an input hazy image. As such, it should not only remove haze as much as possible, but also preserve content and detailed information of origin image. Several prior works have demonstrated that dense connections have the potential to efficiently leverage useful features from different layers and guarantee better convergence via connecting all layers [31,33]. Motivated by this, we design a densely connected structure as the generator, which is able to maximize the information flow from shallow layers to deep layers and enhance reusability of features.

**Figure 2:**
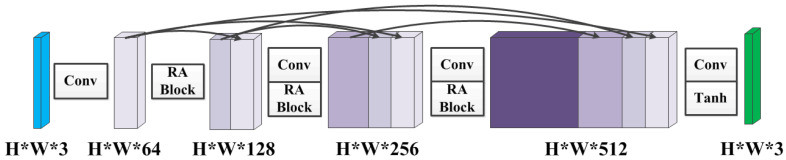
The densely connected structure as the generator. Each “Conv” contains sequence Conv-BN-ReLU, “Tanh” contains sequence Conv-Tanh, and “RA Block” refers to the residual spatial and channel attention module. “Conv” denotes the convolution, “BN” denotes the batch normalization, “ReLU” denotes the rectified linear unit, and “Tanh” denotes an hyperbolic tangent function. The kernel size of each convolution operation is 3×3, the stride is 1×1, and the padding is 1×1. The input and output channel numbers can be obtained according to the parameters in the figure.

As shown in Figure 2, firstly we perform the convolution operation with 64 output channels on the input hazy images for feature dimension expansion. Then for each layer, the feature maps of all preceding layers are utilized as inputs, and its own feature maps are utilized as additional inputs into all subsequent layers. The feature maps are combined through concatenating to ensure direct connections from shallow layers to deep layers. As such, each layer has direct access to back propagation gradients derived from loss functions, thus making the training process much easier. In addition, the residual spatial and channel attention module is designed to improve feature representation and flexibility by taking advantage of both spatial-wise and channel-wise features for robust image dehazing, which is discussed in detail in the next subsection.

### 3.3. Residual Spatial and Channel Attention Module

If the network treats spatial-wise and channel-wise features equally, it would spend plenty of computing power on less effective features, thus greatly limiting the representation of deep neural network [34,35]. In order to make the network concentrate on more informative components and enhance representation of features, we exploit interdependencies among spatial and channel features, resulting in the residual spatial and channel attention module.

**Figure 3:**
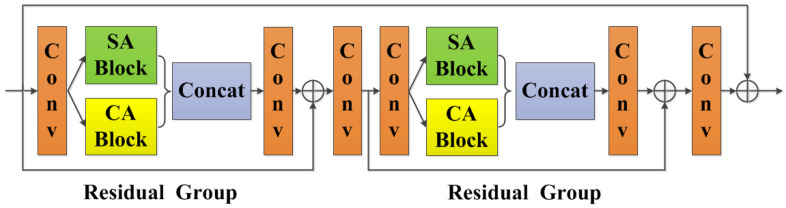
The residual spatial and channel attention module. Each “Conv” contains sequence Conv-BN-ReLU, “SA Block” refers to spatial attention block, and “CA Block” refers to channel attention block, “Concat” refers to concatenation. The kernel size of each convolution operation is 3×3, the stride is 1×1, and the padding is 1×1. Let *C* represent the channel number of the input feature maps, then the input and output channel numbers of 1st, 3rd, 4th and 6th convolution operations are *C*, and the input and output channel numbers of 2nd and 5th convolution operations are 2×C and *C*, respectively.

As shown in Figure 3, the residual spatial and channel attention module consists of two residual groups, a long skip connection and a series of Conv-BN-ReLU operations, since continuous residual groups increase the depth and representation of neural network. Each residual group contains a spatial attention block, a channel attention block, a short skip connection and a series of Conv-BN-ReLU operations. The residual learning is applied to increase the accuracy of image dehazing problems and ease the training of deep neural networks. Skip connections are introduced to capture more useful information instead of simply concatenating feature maps, thus allowing less important information to be bypassed through residual connection. Under such circumstances, the main network would focus on effective information and adaptively learn feature weights from the module, paying more attention to informative regions while retaining content and detailed characteristics. It is worth noting that the residual spatial and channel attention module does not change the width, depth and the number of channels of the input feature maps.

**Figure 4:**
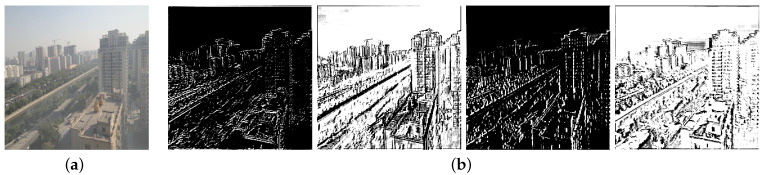
Samples of feature maps: (**a**) input image; (**b**) feature maps extracted from (**a**).

Different feature maps focus on different features in an image. For instance, some feature maps extract texture information from an image, while others extract edge or contour information, as shown in Figure 4. Therefore, it is necessary to treat each feature layer unequally so as to give full play to the representation ability of deep neural network. Motivated by [36], we adopt squeeze-and-excitation block to perform feature recalibration as channel attention mechanism.

**Figure 5:**
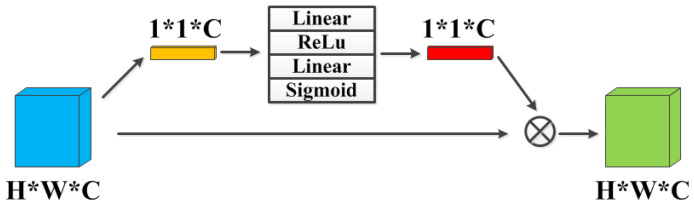
CA Block: The channel attention module. “Linear” and “Sigmoid” denote the linear and sigmoid function, respectively.

As shown in Figure 5, firstly the features are passed through a squeeze operation by global average pooling to generate channel-wise statistics. This allows information aggregated from global receptive field of each feature map to be shared by all layers. Then the aggregation is followed by an excitation operation by employing a simple gating mechanism with Linear-ReLu-Linear-Sigmoid sequence. The function is able to learn a nonlinear interaction between channels to fully capture channel-wise dependencies. Finally, the responses of each feature layer are adaptively recalibrated by explicitly modeling interdependencies between channels. It is worth noting that the channel attention module does not change the width, depth and the number of channels of the input feature maps.

Since channel attention extracts channel-wise statistics among channels, we introduce another complementary block namely spatial attention module with the goal of explicitly modeling interdependencies between pixels for robust dehazing, based on the observation that haze is usually unevenly distributed across an image. Such spatial attention mechanism allows the network to selectively emphasize informative pixels and suppress less useful ones, thus enhancing discriminative learning ability.

**Figure 6:**
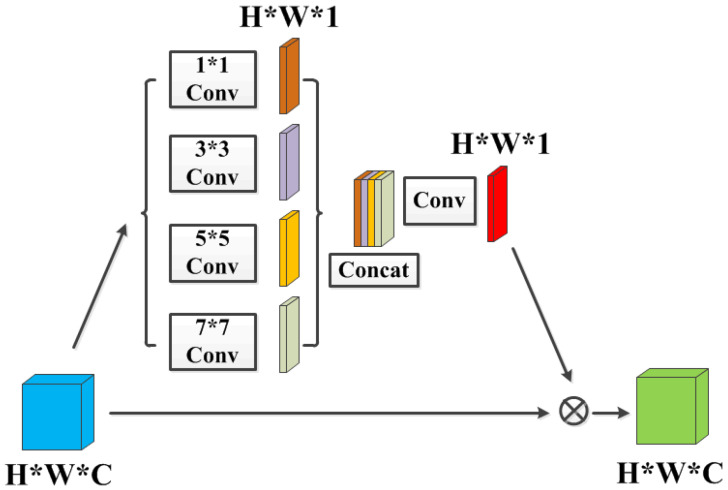
SA Block: The spatial attention module. Each “Conv” contains sequence Conv-BN-ReLU, “Concat” refers to concatenation. The kernel size of the last convolution operation is 3×3, the stride is 1×1, and the padding is 1×1. The input and output channel numbers are 4 and 1, respectively.

As shown in Figure 6, firstly multi-scale convolutions with kernel sizes of 1, 3, 5 and 7 are implemented on input feature maps for feature extraction and dimension reduction. The input and output channel numbers of the multi-scale convolutions are *C* and 1, respectively. Convolutions of different scales are capable of providing different receptive fields, while preserving effective details of features on various scales [37,38]. Then these reduced features are concatenated together followed by sequence Conv-BN-ReLU for dimension reduction. Finally, pixel-wise multiplication between input feature maps and spatial attention map with adaptively learning weights is implemented, thus leading the network to be more focused on informative pixels. Therefore, the spatial-wise and channel-wise features are complementary to each other to achieve more visually pleasant perception. It is worth noting that the spatial attention module does not change the width, depth and the number of channels of the input feature maps.

### 3.4. Discriminator

**Figure 7:**
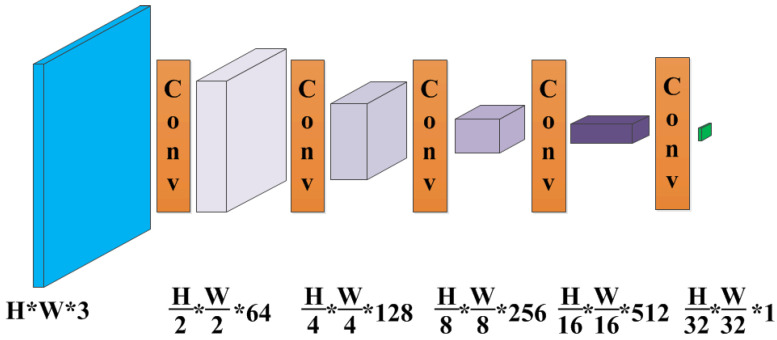
The PatchGAN architecture as the discriminator. Each “Conv” contains sequence Conv-BN-ReLU. The kernel size of each convolution operation is 3×3, the stride is 2×2, and the padding is 1×1. The input and output channel numbers can be obtained according to the parameters in the figure.

The goal of the discriminator is to distinguish whether the restored image is hazy or clear. As shown in Figure 7, we adopt the same PatchGAN architecture as described in pix2pix [32], which only penalizes structure at the scale of patches. PatchGAN is a full convolution network, and performs patch-wise comparison instead of pixel-wise comparison between the reconstructed image and target image. The effective receptive field of the output matrix is larger than a pixel, since it covers a patch of the image. This is beneficial to preserve texture information and remove artifacts in the image. Finally, the ultimate result is an average of the values in output matrix.

### 3.5. Loss Function

Since GANs are known to be unstable during training and may introduce artifacts in the output image, we adopt a multi-loss function which consists of adversarial loss, L1 loss, contrastive loss and registration loss to train the proposed network. In what follows, we elaborate these losses in detail.

#### 3.5.1. Adversarial Loss

The objective of adversarial loss is to learn the data distribution of target domain to synthesize a clear image from an input hazy image. In this paper, we apply conditional GAN where the generator learns to generate a mapping function with a conditional variable. The adversarial loss can be expressed as

(3) $$L_{adv}=E_{x,y}\left[\log D\left(x,y\right)\right]+E_{x,z}\left[\log\left(1-D\left(x,G\left(x,z\right)\right)\right)\right],$$

where *x* denotes the input hazy image, *y* denotes the corresponding haze-free image and *z* denotes the noise. We follow the method used in pix2pix [32], where the noise is applied in the form of dropout at both training and test time. The generator *G* aims to minimize this objective while the discriminator *D* tries to maximize it. To achieve this, we perform one generator update, followed by one discriminator update alternatively.

#### 3.5.2. L1 Loss

Previous studies have found that it is beneficial to mix the adversarial loss with L1 loss for fewer artifacts and less color distortion, where L1 loss encourages pixel-level consistency between the restored haze-free image and ground truth. L1 loss can be expressed as

(4) $$L_{L1}=E_{x,y,z}[||y-G\left(x,z\right){\left|\right|}_{1}].$$

#### 3.5.3. Contrastive Loss

Most existing learning based dehazing networks only adopt corresponding clear images during training to back propagate the gradients, while hazy images are only utilized as input to the network. Inspired by contrastive learning [39] which aims to learn a representation by comparing the data with positive samples and negative samples in the feature space, we refine a novel pixel-wise contrastive loss by exploiting both hazy images and clear images to generate better dehazed images.

There are two aspects that need to be considered: One is how to construct positive pairs and negative pairs, and the other is how to build feature representation space for contrast. As described in [40], the positive pairs consist of the restored image and corresponding clear image, while the negative pairs comprise the restored image and corresponding hazy image. For simplicity, we represent hazy image, restored image and clear image as negative, anchor and positive, respectively. Contrastive learning aims to pull anchor closer to positive, and push anchor far away from negative in the representation space.

**Figure 8:**
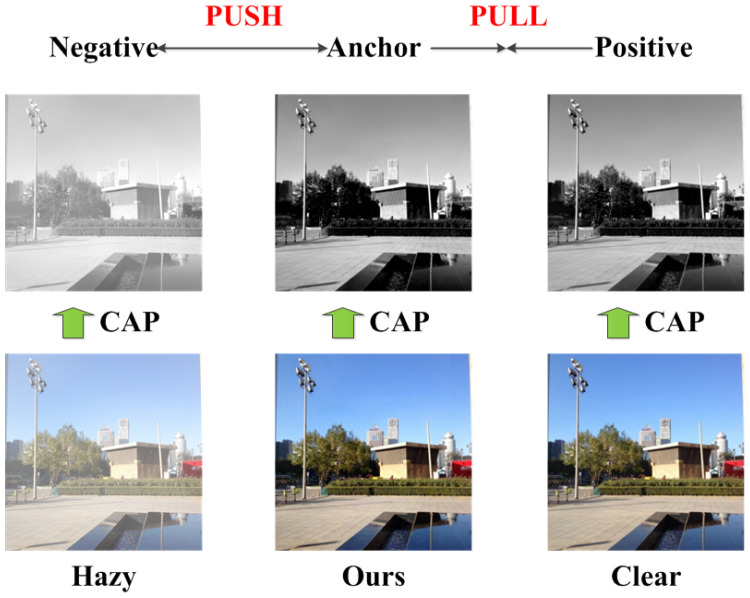
The diagram of contrastive learning. “CAP” denotes the process of obtaining concentration of haze based on color attenuation prior.

According to the color attenuation prior [11], the concentration of haze is positively correlated to the difference between brightness and saturation of an image, since hazy regions are often characterized with high brightness and low saturation [41]. To utilize this prior, we implement contrastive learning on the color attenuation representation space, so as to make the restored dehazed images approximate clear images and move away from hazy images in terms of hazy concentration across an image. The diagram of contrastive learning is presented in Figure 8, which is able to promote detail restoration and haze removal. As expected, denser haze results in larger brightness, lower saturation and higher difference between brightness and saturation. The contrastive loss is refined as [42]

(5) $$L_{con}=E_{x,y,z}{\left[||f\left(y\right)-f\left(G\left(x,z\right)\right){\left|\right|}_{1}-||f\left(x\right)-f\left(G\left(x,z\right)\right){\left|\right|}_{1}+||f\left(y\right)-{f\left(x\right)||}_{1}\right]}_{+},$$

where *f* denotes the concentration of haze, which is defined as the difference between brightness and saturation of the image.

#### 3.5.4. Registration Loss

As discussed earlier, GANs may introduce artifacts and produce noisy results, which inevitably makes recovered images visually unpleasant. To address this issue, we propose a novel refined registration loss to measure the visual difference between the restored image and counterpart haze-free image by leveraging scale invariant feature transform (SIFT) feature detection [43] and feature matching, which is beneficial to restoring details and generating visually pleasing results.

**Figure 9:**
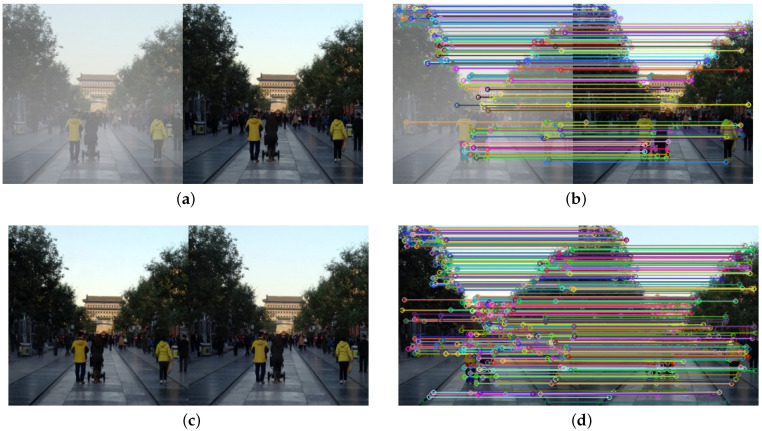
The schematic diagram of image registration: (**a**) the hazy and corresponding haze-free images; (**b**) image registration of (**a**); (**c**) the restored and corresponding haze-free images; (**d**) image registration of (**c**).

Image registration is the process of mapping and geometrically aligning two images [44,45]. In this paper, we employ two important steps of image registration process, namely feature detection and feature matching, to construct the registration loss. First, we apply the widely used SIFT algorithm to extract feature points, which is invariant to rotation, translation and scale changes. Then we adopt the nearest-neighbor method to find matching points of two input images based on Euclidean distance. However, there may exist some errors after Euclidean distance initial matching because of the influence from background clutter or detection error. To solve this, random sample consensus (RANSAC) algorithm is performed to filter out mismatches between point pairs and improve accuracy [46]. The schematic diagram of image registration between hazy and corresponding haze-free images, and between restored and corresponding haze-free images is present in Figure 9.

As expected, larger similarity results in greater number of matching points and smaller Euclidean distance between them. Motivated by this, we employ average Euclidean distance of SIFT eigenvectors as the judgment basis of similarity of matching feature points in two images. Thus the registration loss is defined as

(6) $$L_{reg}=E_{x,y,z}[\frac{1}{N}\sum_{i=1}^{N}\left|\right|h_{i}\left(y\right)-h_{i}\left(G\left(x,z\right)\right){\left|\right|}_{E}],$$

where *N* is the number of matching point pairs and $h_{i}$ denotes the *i*-th 128-dimensional SIFT eigenvectors of matching points. The main idea of this loss is to compare the restored image with haze-free image in a multi-dimensional feature space rather than a pixel space, aiming to reinforce fine features and preserve detailed information.

#### 3.5.5. Total Loss

Specifically, we combine adversarial loss, L1 loss, contrastive loss and registration loss together with appropriate weights to form total loss function. The total loss can be formulated as follows:

(7) $$\begin{array}{c} L_{all}=L_{adv}+\lambda_{L1}L_{L1}+\lambda_{con}L_{con}+\lambda_{reg}L_{reg}, \end{array}$$

where $\lambda_{L1}$, $\lambda_{con}$ and $\lambda_{reg}$ are the trade-off parameters.

## 4. Experimental Results

In this section, we conducted experiments on both synthetic datasets and real-world images to evaluate our proposed densely connected residual spatial and channel attention network. We compare our proposed architecture with the following state-of-the-art dehazing methods: DCP [10], CAP [11], AODNet [15], EPDN [47], GCANet [48], pix2pix [32], FFA-Net [18] and Two-branch [49]. Moreover, ablation studies are presented to demonstrate the effectiveness of the proposed module and loss functions.

### 4.1. Datasets

We conducted experiments on the publicly available Realistic Single Image Dehazing (RESIDE) dataset [50], which is a large-scale dataset consisting of both synthetic and real-world hazy images for fairly evaluation and comparison. The atmospheric scattering model was applied where the global atmospheric light is randomly selected between (0.7, 1.0) for each channel, and the scattering coefficient is randomly chosen between (0.6, 1.8). For training, we selected 2000 hazy and corresponding haze-free images from the Outdoor Training Set (OTS), which contains paired clean outdoor images and generated hazy ones with different parameters. For testing on synthetic images, we choose 300 synthetic hazy images from the Synthetic Objective Testing Set (SOTS), in which the hazy images are synthesized following the same process as training data. For testing on real-world images, 10 hazy images from the Hybrid Subjective Testing Set (HSTS), which are collected from real-world outdoor scenes, and 200 hazy images from the Real-world Task-driven Testing Set (RTTS), which covers mostly traffic and driving scenarios, are provided.

In addition, we implement our method on real-world dehazing benchmarks with the O-HAZE dataset [51] utilized in NTIRE2018 Dehazing Challenge [52], DENSE-HAZE dataset [53] utilized in NTIRE2019 Dehazing Challenge [54] and NH-HAZE dataset [55] utilized in the NTIRE2020 Dehazing Challenge [56]. O-HAZE, DENSE-HAZE and NH-HAZE contain 45 outdoor hazy images, 55 dense hazy images and 55 nonhomogeneous hazy images with their corresponding ground truth, respectively. The datasets were captured in presence or absence of haze in various scenes using a professional haze generator that imitates the real conditions of haze scenes. Among these 155 pairs of images, 140 pairs were utilized to train our proposed models, and the remaining pairs were used for testing.

### 4.2. Implementation Details

We employed the PyTorch framework with NVIDIA GEFORCE RTX 3090 TI GPU on both training and testing stages. Images were resized to 256 × 256 through preprocessing, and the ADAM optimizer was implemented with a batch size of 1. The proposed network was trained with a total of 200 epochs for convergence, in which the learning rate was set to be 0.0001 for the former 100 epochs and reduced linearly to 0 during the latter 100 epochs. The trade-off weights were empirically set to be $\lambda_{L1}=80$, $\lambda_{con}=40$ and $\lambda_{reg}=0.03$. The runtime for one image on the RTX 3090 TI GPU was about 0.143 s on average.

### 4.3. Experiments on Synthetic Images

**Figure 10:**
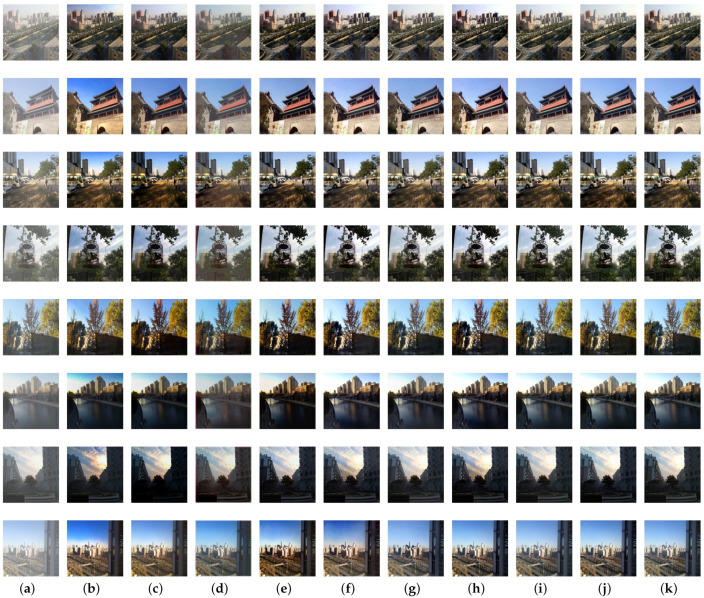
Visual results on synthetic images of RESIDE dataset: (**a**) hazy images; (**b**) DCP; (**c**) CAP; (**d**) AODNet; (**e**) EPDN; (**f**) GCANet; (**g**) pix2pix; (**h**) FFA-Net; (**i**) Two-branch; (**j**) our proposed method; (**k**) corresponding haze-free images.

To better demonstrate the effectiveness of our proposed network, we first conducted experiments on the synthetic objective testing set compared with other state-of-the-art methods. The quantitative results in terms of PSNR and SSIM metrics [57] are given in Table 1, and the visual comparisons are provided in Figure 10. Furthermore, to evaluate perceptual quality, we also introduce the perceptual index (PI) [58] as a criterion in Table 1. PI bridges the visual effect with computable index, which can be formulated as

(8) $$\begin{array}{c} PI=\frac{1}{2}\left(\left(10-Ma\right)+NIQE\right), \end{array}$$

where Ma [59] and NIQE [60] are two image qualification indexes. It is observed that our proposed densely connected residual spatial and channel attention network achieves the best PSNR and SSIM results, and attains the gain with 1.8935 dB in PSNR and 0.0135 in SSIM compared with the efficient FFA-Net architecture. PI measures the quality of recovered images based on human perception, and a lower PI indicates better perceptual quality. We also observe that the proposed method achieves a competitively low PI score. As shown in Figure 10, DCP and CAP suffer from color distortion especially in the sky region. AODNet fails to remove haze thoroughly and the restored images are not clear enough. EPDN sometimes generates darker images compared with the corresponding haze-free images. The detailed and texture features are fuzzy in the images recovered from GCANet. It can be clearly observed that there exists some artifacts introduced by pix2pix. FFA-Net reconstructs haze-free images without sharper structures. The details of the recovery by the two-branch method are sometimes not particularly clear. Compared with above methods, our proposed architecture generates better visually haze-free images and effectively preserves color and texture information, improves the dehazing results both quantitatively and qualitatively.

### 4.4. Experiments on Real-World Images

**Figure 11:**
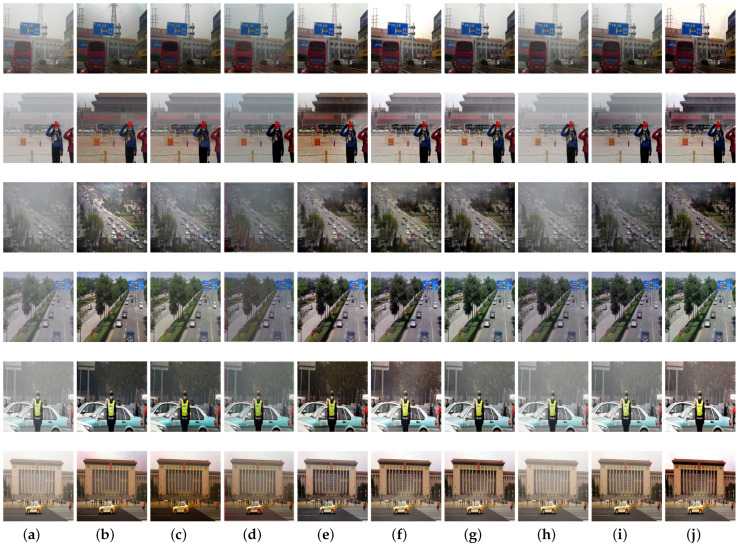
Visual comparisons on real-world images. (**a**) Hazy images. (**b**) DCP. (**c**) CAP. (**d**) AODNet. (**e**) EPDN. (**f**) GCANet. (**g**) pix2pix. (**h**) FFA-Net. (**i**) Two-branch. (**j**) Our proposed method.

Figure 11 depicts the visual comparisons on real-word images provided by hybrid subjective testing set and real-world task-driven testing set. We observe that DCP generates unsatisfactory dehazing results with darker images, and CAP is not able to remove haze thoroughly. The effect of haze removal by AODNet is not obvious. EPDN produces color artifacts in some hazy regions while removing haze. GCANet recovers haze-free images without sharper structures and details. The reconstructed images by pix2pix is fuzzy without sharper edges. FFA-Net suffers from undesirable dehazing effect and fails to generate ideal haze-free results for this set of images. Two-branch method sometimes is not able to remove haze thoroughly and may cause color distortion to some extent. Compared with these methods, our proposed architecture generates more natural and realistic dehazed results with fewer artifacts and less color distortion, which demonstrates the effectiveness of our proposed network.

**Figure 12:**
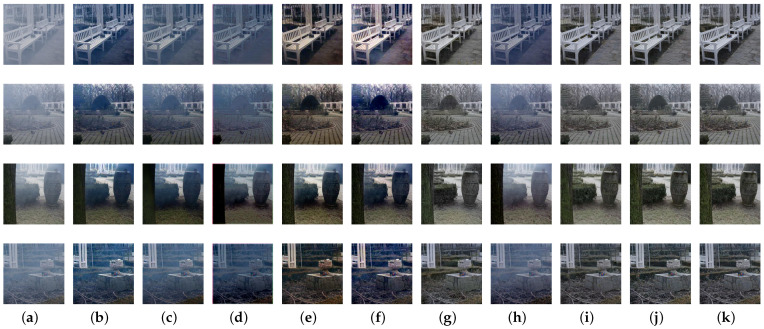
Visual comparisons on the NTIRE dehazing challenge datasets: (**a**) hazy images; (**b**) DCP; (**c**) CAP; (**d**) AODNet; (**e**) EPDN; (**f**) GCANet; (**g**) pix2pix; (**h**) FFA-Net; (**i**) two-branch; (**j**) our proposed method; (**k**) corresponding haze-free images.

We also evaluate the proposed method on the NTIRE dehazing challenge datasets. The quantitative comparisons are present in Table 2, and the visual results are given in Figure 12. We observe that the effect of haze removal is not obvious for DCP, CAP and AODNet methods. EPDN achieves certain dehazing effect but also brings serious color distortion. GCANet achieves the effect of local area image dehazing with color artifacts. Pip2pix reconstructs quite well but some recovered details are fuzzy. The dehazing effect of FFA-Net is also not obvious, and it is not suitable for this kind of scenes. The two-branch method presents good performance of image dehazing with vivid color in these kind of data and obtains the best PSNR and SSIM scores. The proposed method generates visually better haze-free images with the second best PSNR and SSIM scores and a competitive PI score. Although some details may no be clear enough as the ground truth, the proposed framework has a great potential and performs in general better than some other considered methods.

**Figure 13:**
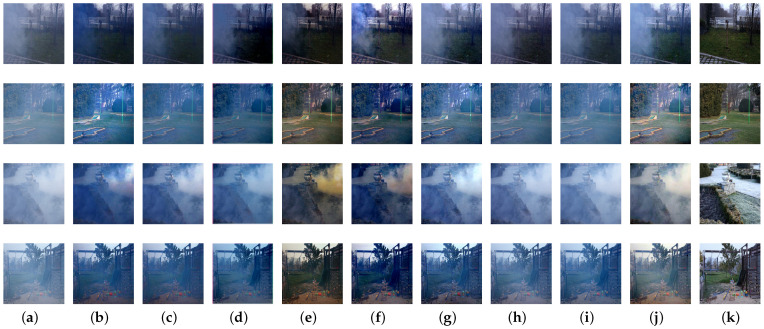
Visual results on the NTIRE dehazing challenge datasets with the network training on RESIDE datasets: (**a**) hazy images; (**b**) DCP; (**c**) CAP; (**d**) AODNet; (**e**) EPDN; (**f**) GCANet; (**g**) pix2pix; (**h**) FFA-Net; (**i**) two-branch; (**j**) our proposed method; (**k**) corresponding haze-free images.

Furthermore, to investigate the effectiveness of our proposed framework, we conducted another experiment on the NTIRE dehazing challenge datasets, in which the network is trained with RESIDE datasets instead of NTIRE dehazing challenge images. The quantitative results are given in Table 3, and the visual comparisons are presented in Figure 13. We observe that the dehazing performance of almost all methods drops drastically on the test NTIRE dehazing challenge dataset that follows different distribution to the training RESIDE dataset. The dense haze regions are almost poorly handled and the dehazing effect is not visually obvious. However, compared with other methods, our network achieves certain dehazing effect in some areas, preserves structural and detailed properties without introducing severe color distortion, which demonstrates the potential of our proposed architecture in terms of image dehazing. Moreover, it is also observed that for learning-based image dehazing networks, the dehazing performance is strictly influenced by the training data.

### 4.5. Ablation Study

**Figure 14:**
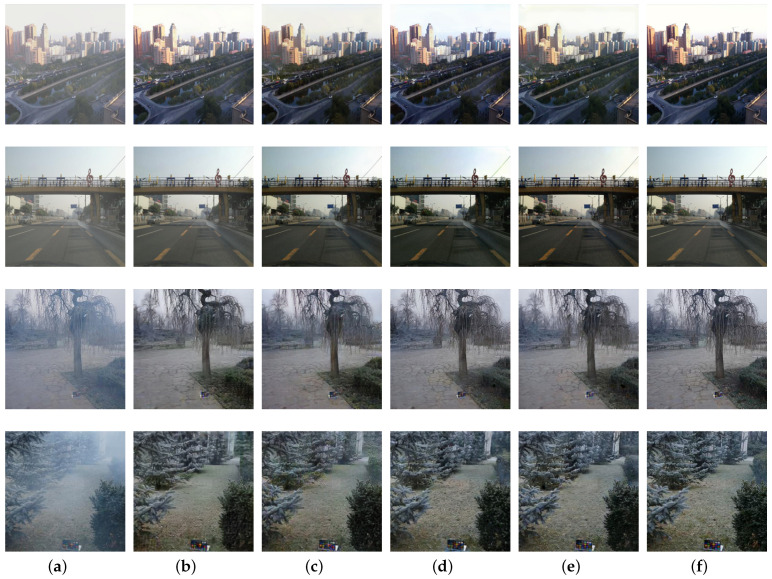
Visual comparisons with different attention modules: (**a**) hazy images; (**b**) RG; (**c**) RG+CA; (**d**) RG+SA; (**e**) RG+CA+SA; (**f**) corresponding haze-free images.

To better verify the effectiveness of our proposed architecture, a series of ablation studies were implemented for analysis. Firstly, we constructed the following residual groups with different attention modules: (1) RG: residual group with no attention block; (2) RG+CA: residual group with channel attention block; (3) RG+SA: residual group with spatial attention block; (4) RG+CA+SA: residual group with both channel and spatial attention blocks. The quantitative comparisons are presented in Table 4 and Table 5, and the visual results are given in Figure 14. We observe that integrating channel and spatial attention module attains higher PSNR and SSIM results with visually better haze-free images. Channel and spatial blocks are implemented to capture channel-wise and spatial-wise dependencies for robust dehazing, with which the subsequent networks would pay more attention to effective feature maps and informative pixels, thus leading to vivid colors and increased contrast.

**Figure 15:**
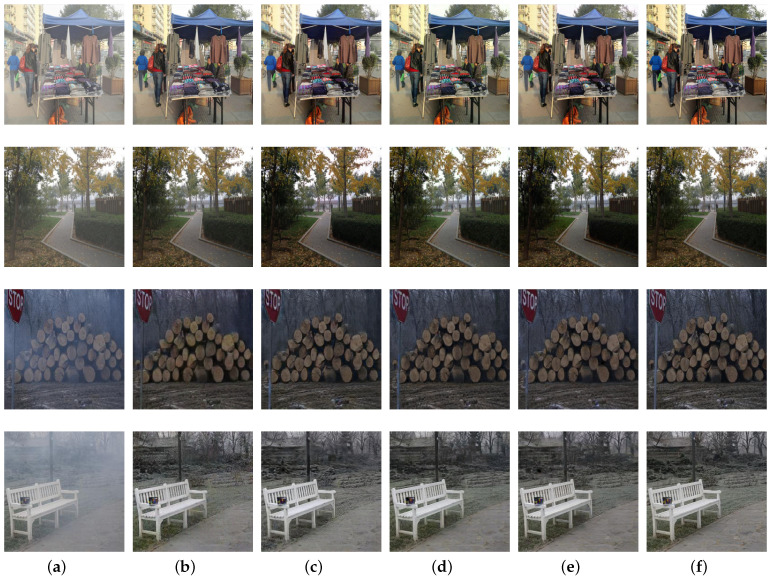
Visual results with different loss functions: (**a**) hazy images; (**b**) without both losses; (**c**) without registration loss; (**d**) without contrastive loss; (**e**) with both losses; (**f**) corresponding haze-free images.

To demonstrate the effectiveness of loss functions proposed in this paper, the network was trained without contrastive loss or registration loss. The quantitative results are provided in Table 6 and Table 7, and the visual comparisons are presented in Figure 15. The restored images with both contrastive loss and registration loss achieve the best PSNR and SSIM results. In addition, registration loss contributes to restoring sharper structures and detailed information, while contrastive loss is beneficial to removing haze completely.

## 5. Conclusions

In this paper, inspired by the significant performance of conditional generative adversarial framework, we propose an end-to-end trainable densely connected residual spatial and channel attention network for single image dehazing, which does not estimate intermediate atmospheric scattering parameters. Specifically, we propose a novel residual spatial and channel attention module, which adaptively recalibrates spatial-wise and channel-wise feature responses by considering interdependencies among spatial and channel information. Furthermore, contrastive loss and registration loss are proposed to restore sharper structures and generate visually better haze-free images. Experimental results on both synthetic and real-world datasets have shown that the proposed method has achieved the state-of-the-art results. Through ablation studies, we studied the effectiveness of different factors on the performance of proposed architecture.

## Figures and Tables

**Table 1:** Quantitative comparisons with other methods on synthetic images.

Metrics	DCP	CAP	AODNet	EPDN	GCANet	pix2pix	FFA-Net	Two-Branch	Ours
**PSNR**	17.4582	18.3581	19.7542	21.3050	23.4265	26.9524	31.0752	32.8842	32.9660
**SSIM**	0.8752	0.8102	0.8697	0.8793	0.9124	0.9283	0.9548	0.9680	0.9683
**PI**	2.7793	2.9076	3.0314	2.8232	2.8774	2.7966	2.7861	2.7912	2.7927

**Table 2:** Quantitative results on the NTIRE dehazing challenge datasets.

Metrics	DCP	CAP	AODNet	EPDN	GCANet	pix2pix	FFA-Net	Two-Branch	Ours
**PSNR**	17.9749	17.0929	17.1099	17.1335	17.9412	18.4239	17.6025	19.5301	19.2072
**SSIM**	0.6958	0.6546	0.6174	0.7013	0.7258	0.7334	0.6890	0.7624	0.7454
**PI**	2.9803	3.4075	3.5217	2.8029	2.8139	2.7451	3.2858	2.8142	2.7874

**Table 3:** Quantitative comparisons on the NTIRE dehazing challenge datasets with the network training on RESIDE datasets.

Metrics	DCP	CAP	AODNet	EPDN	GCANet	pix2pix	FFA-Net	Two-Branch	Ours
**PSNR**	13.0425	12.6594	12.6873	13.3428	13.4772	13.1627	12.7565	12.7378	13.6301
**SSIM**	0.5162	0.4828	0.5047	0.5583	0.5669	0.5311	0.5343	0.5427	0.5896
**PI**	4.3941	5.2311	5.3762	4.3171	3.9761	4.5535	5.2122	5.8446	4.4889

**Table 4:** Quantitative comparisons with different attention module on RESIDE dataset.

Metrics	RG	RG+CA	RG+SA	RG+CA+SA
**PSNR**	28.9251	31.1037	31.3245	32.9660
**SSIM**	0.9328	0.9523	0.9583	0.9683
**PI**	2.7469	2.8667	2.7142	2.7927

**Table 5:** Quantitative results with different attention module on NTIRE dehazing challenge datasets.

Metrics	RG	RG+CA	RG+SA	RG+CA+SA
**PSNR**	18.1372	18.5522	18.8035	19.2072
**SSIM**	0.7228	0.7366	0.7262	0.7454
**PI**	2.6952	2.7913	2.7795	2.7874

**Table 6:** Quantitative results with different loss functions on RESIDE dataset. ’wob’, ’wor’, ’woc’ and ’wb’ denote without both losses, without registration loss, without contrastive loss and with both losses, respectively.

Metrics	Wob	Wor	Woc	Wb
**PSNR**	30.3453	32.0425	31.9728	32.9660
**SSIM**	0.9482	0.9581	0.9561	0.9683
**PI**	2.7825	2.8143	2.7682	2.7927

**Table 7:** Quantitative comparisons with different loss functions on NTIRE dehazing challenge datasets. ’wob’, ’wor’, ’woc’ and ’wb’ denote without both losses, without registration loss, without contrastive loss and with both losses, respectively.

Metrics	Wob	Wor	Woc	Wb
**PSNR**	18.6476	18.9427	18.8752	19.2072
**SSIM**	0.7392	0.7424	0.7416	0.7454
**PI**	2.7351	2.7477	2.7895	2.7874

## Data Availability

The RESIDE dataset, O-HAZE dataset, DENSE-HAZE dataset, and NH-HAZE dataset are made publicly available for research purposes. For more information, please refer to the websites https://sites.google.com/site/boyilics/website-builder/reside/, https://data.vision.ee.ethz.ch/cvl/ntire18//o-haze/, https://data.vision.ee.ethz.ch/cvl/ntire19//dense-haze/, and https://data.vision.ee.ethz.ch/cvl/ntire20//nh-haze/ (accessed on 23 November 2021).
